# Enriched Riceberry Bran Oil Exerts Chemopreventive Properties through Anti-Inflammation and Alteration of Gut Microbiota in Carcinogen-Induced Liver and Colon Carcinogenesis in Rats

**DOI:** 10.3390/cancers14184358

**Published:** 2022-09-07

**Authors:** Warunyoo Phannasorn, Aroonrat Pharapirom, Parameth Thiennimitr, Huina Guo, Sunantha Ketnawa, Rawiwan Wongpoomchai

**Affiliations:** 1 Department of Biochemistry, Faculty of Medicine, Chiang Mai University, Chiang Mai 50200, Thailand; 2 Department of Microbiology, Faculty of Medicine, Chiang Mai University, Chiang Mai 50200, Thailand

**Keywords:** anti-inflammation, anti-carcinogenicity, bioactive food components, cancer prevention, gut microbiota, Riceberry, rice bran oil, short-chain fatty acids

## Abstract

**Simple Summary:**

Rice bran oil is gaining popularity around the world due to its ability to improve lipid profiles. Recent in vitro studies have shown that the active compounds in colored rice bran oil exhibited anti-cancer properties in various cell lines. However, there has been a limited number of animal studies focusing on the anti-carcinogenic action of rice bran oil. In this study, Riceberry bran oil (RBBO) extracted from the bran of a Thai-pigmented rice variety, namely Riceberry, was investigated for its inhibitory mechanism on the early stages of liver and colorectal carcinogenesis using the dual carcinogens-induced rat model. RBBO was able to inhibit the biomarkers of rat liver cancer and colon cancer by forcing cells to undergo apoptosis, reducing inflammation, and changing the profiles of bacteria and their metabolites. These findings suggest that RBBO could be a promising source of high-value chemopreventive agents in terms of both cancer prevention and treatment.

**Abstract:**

Riceberry has recently been acknowledged for its beneficial pharmacological effects. Riceberry bran oil (RBBO) exhibited anti-proliferation activity in various cancer cell lines. However, animal studies of RBBO on anti-carcinogenicity and its molecular inhibitory mechanism have been limited. This study purposed to investigate the chemopreventive effects of RBBO on the carcinogen-induced liver and colorectal carcinogenesis in rats. Rats were injected with diethylnitrosamine (DEN) and 1,2-dimethylhydrazine (DMH) and further orally administered with RBBO equivalent to 100 mg/kg body weight of γ-oryzanol 5 days/week for 10 weeks. RBBO administration suppressed preneoplastic lesions including hepatic glutathione *S*-transferase placental form positive foci and colorectal aberrant crypt foci. Accordingly, RBBO induced hepatocellular and colorectal cell apoptosis and reduced pro-inflammatory cytokine expression. Interestingly, RBBO effectively promoted the alteration of gut microbiota in DEN- and DMH-induced rats, as has been shown in the elevated *Firmicutes*/*Bacteroidetes* ratio. This outcome was consistent with an increase in butyrate in the feces of carcinogen-induced rats. The increase in butyrate reflects the chemopreventive properties of RBBO through the mechanisms of its anti-inflammatory properties and cell apoptosis induction in preneoplastic cells. This would indicate that RBBO containing γ-oryzanol, phytosterols, and tocols holds significant potential in the prevention of cancer.

## 1. Introduction

Cancer is one of the leading causes of death worldwide with nearly 10 million deaths in 2020 reported by the World Health Organization. Colorectal and liver cancer are ranked as the second and third most common types of cancer resulting in death. Obesity, an unhealthy diet, a lack of exercise, smoking, and alcohol consumption are important risk factors for cancer. Cancer can result from interactions between an individual genetic factor and certain external agents, such as carcinogens, resulting in the transformation of normal cells into tumor cells [1]. Therefore, an intervention of the mutation and proliferation of uncommon cells is a key objective in inhibiting carcinogenesis.

At present, nutraceuticals have garnered significant attention for their nutritional value and protective capabilities against disease. They exhibit the potential to treat a variety of illnesses that include diabetes, atherosclerosis, cardiovascular disease, cancer, and neurological disorders [2]. Vegetable oil has been recommended for use in daily cooking due to its high contents of monounsaturated fatty acids (MUFAs) and polyunsaturated fatty acids (PUFAs), as well as for the positive health benefits. It has been found to exert on heart disease and cancer in clinical studies [3,4]. PUFAs and MUFAs are known to be able to reduce the risk of cardiovascular disease and lower cholesterol levels in obese patients [5]. The consumption of MUFAs that have been derived from plants, particularly olive oil, has been linked to a decreased risk of developing cancer [6]. Similarly, the substitution of plant-based MUFAs for animal-based MUFAs has been associated with a lower number of cancer deaths [7]. The effect of PUFAs on cancer risk is directly proportional to the ratio of ω-6 to ω-3 PUFAs. The 4:1 ratio of ω-6 to ω-3 has been indicated in reducing inflammation, which has been implicated as a risk factor for various chronic conditions [8]. Some documented evidence has suggested that ω-6 PUFAs could stimulate tumor development, whereas ω-3 PUFAs could protect against tumor formation. The administration of a diet rich in ω-6 given to mice resulted in increased cyclooxygenase-2 (COX-2) levels and expanded epigenetic activation of prostaglandin-endoperoxide synthase-2. These changes increased the production of prostaglandin E2 from arachidonic acid in conjunction with the gene silencing that is associated with a number of tumor protective factors. They were also observed to increase the presence of adenomatous polyposis coli and the accumulation of the factors involved in cell proliferation (*Ccnd1*). Furthermore, these changes enhanced oncogenic transformation (*c-JUN*), which may contribute to colonic inflammation and the progression of cancer [9]. On the other hand, a diet that is rich in ω-3 PUFAs inhibited the formation of MC38 colorectal cancer in mice, while the treatment of tumors with epoxydocosapentaenoic acids and ω-3 PUFAs metabolites was found to decrease proto-oncogenes expression in tumor tissues [10].

Commensal bacteria in the gastrointestinal tract serve a number of critical roles including epithelial formation, host metabolism, pathogenic defense, and immunological regulation. By contrast, dysbiosis refers to the altered composition and function of gut microbiota leading to the development of a variety of pathological diseases. This is particularly true in inflammatory bowel disease (IBD), certain IBD-associated cancers, and hepatocellular carcinoma [11]. Dietary lipids impact the microbiome that may be advantageous or detrimental to the host. Changes in the gut microbiota in mice fed a high-fat diet (HFD) enriched with saturated fat were associated with increased intestinal ROS generation and oxidative stress [12] which are known to play a crucial role in the development and progression of cancer [13]. HFD promotes colorectal carcinogenesis in both AOM-treated and Apc^min/+^ mice by promoting significant changes in the composition of the gut microbiota that have been associated with increased pathogenic bacteria and reduced probiotic bacteria. Moreover, HFD has been observed to alter gut barrier functions [14]. Furthermore, a high-cholesterol diet can promote hepatocellular carcinoma in mice by increasing the hepatic retention of hydrophobic bile acids caused by dysbiosis [15]. Short-chain fatty acids (SCFA) including acetate, propionate, and butyrate, are bacterial metabolites produced from anaerobic fermentation of non-digestible dietary ingredients in the colon. They play a crucial role in the gut microbiota homeostasis and are involved in the protection of certain chronic diseases [16]. The dietary approach to modulate SCFA levels might serve as a potential chemoprevention.

Rice bran oil is considered a healthy oil due to its fatty acid profile and its unique combination of certain predominantly biologically active ingredients such as γ-oryzanol, tocopherols, tocotrienols, certain unsaponifiable substances, and many phytosterols [17]. Studies on the biological activities of rice bran oil reported effective anti-diabetic, anti-cancer, anti-inflammatory, and hypolipidemic properties [18]. Recently, various studies have reported the potential of Riceberry bran oil (RBBO) to ameliorate hyperglycemia, relevant lipid profiles, and oxidative stress in streptozotocin-induced diabetic rats fed a HFD [19]. Furthermore, it was found to be able to inhibit the proliferation of certain cancer cell lines [20,21]. However, the anti-cancer effect of RBBO in animal models has not yet been fully studied. Many forms of cancer have been linked to a range of environmental carcinogens, particularly those found in contaminated food. These carcinogens have been implicated in incidences of liver and colorectal cancer [22]. The dual organ carcinogenicity test employs both diethylnitrosamine (DEN) and 1,2-dimethylhydrazine (DMH), which are metabolized by the same cytochrome, namely P450, to initiate carcinogenesis in the liver and colorectum, respectively [23,24]. In this study, these carcinogens were administered to the same rats in order to reduce the number of animals included in the experimental procedure, according to the Three Rs principle of animal research [23,24,25]. Therefore, the purpose of this study was to investigate the chemopreventive effects of RBBO in cases of carcinogen-induced liver and colorectal carcinogenesis in rats, as well as to elucidate the relevant mechanisms of action at the molecular level. The important carcinogenesis-promoting factors of RBBO that were associated with its inflammatory condition, as well as those of gut microbiota and its metabolites, were also analyzed.

## 2. Materials and Methods

### 2.1. Chemicals

DEN and metaphosphoric acid were obtained from Sigma-Aldrich (St. Louis, MO, USA), while DMH was obtained from TCI (Tokyo, Japan). An ApopTag^®^ Peroxidase in situ Apoptosis Detection Kit, hydrogen peroxide (H_2_O_2_), methylene blue, and skim milk were purchased from Merck (Darmstadt, Germany). Anti-rat glutathione *S*-transferase placental form (GST-P) was acquired from MBL (Nagoya, Japan). EnvisionTM G/2 Doublestain System Rabbit/Mouse (DAB+/Permanent Red) was bought from Agilent (Santa Clara, CA, USA). Purezol reagent was acquired from Bio-Rad (Hercules, CA, USA). A high-capacity cDNA reverse transcription kit was purchased from Applied Biosystems (Foster City, CA, USA), while a SensiFAST SYBR Lo-ROX Kit was procured from Bioline Reagent Ltd. (London, UK).

### 2.2. RBBO Sample

RBBO was supplied by Kurk Rice Mill (Chiang Rai, Thailand). The extraction process and major chemical constituents have been described in our previously published report [26]. Briefly, RBBO was extracted via the cold pressing method obtaining crude oil and was further purified by press filtration, and various phytochemicals were immediately analyzed by gas chromatography–mass spectrometry, high-performance liquid chromatography, and spectrophotometry. The main fatty acids of RBBO used in this study were composed of 42.61% oleic acid and 30.76% linoleic acid. One gram of RBBO contained 56.74 mg of γ-oryzanol, 6.01 mg of phytosterols, and 1.46 mg of total vitamin E with γ-tocotrienol as a major tocol [26].

### 2.3. Animals and Experimental Protocol

**Figure 1:**
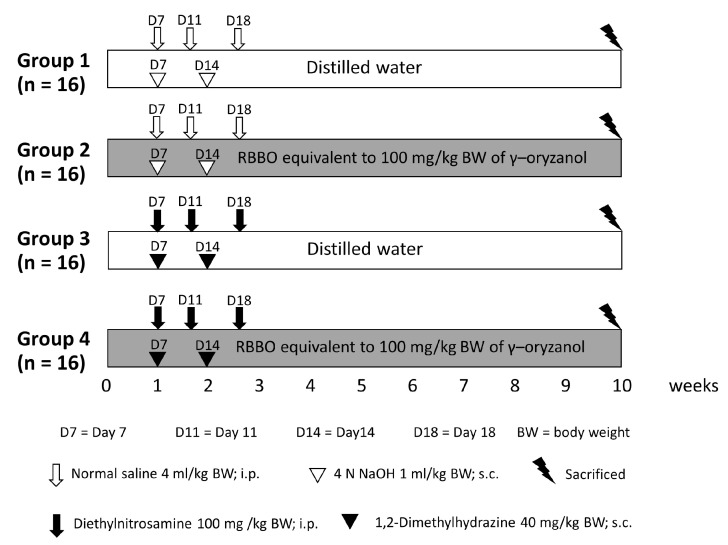
Experimental protocol of RBBO treatment in DEN- and DMH-induced preneoplastic lesions in livers and colons of rats.

Three-week-old male Wistar rats (weighing 60–80 g) were obtained from the National Laboratory Animal Center (Nakhon Pathom, Thailand). They were housed under conventional circumstances at a temperature of 25 °C and by employing a 12-hour dark/12-h light cycle. They were given free access to drinking water and fed standard rodent food. The Animal Ethics Committee of the Faculty of Medicine, Chiang Mai University (41/2561) authorized the experimental procedure employed in this study, as presented in Figure 1. Rats were randomly separated into four groups, wherein 16 rats were placed in each group. Group 1 served as a negative control, while group 3 was representative of a positive control. Groups 2 and 4 were fed the equivalent of 100 mg of γ-oryzanol/kg body weight of RBBO for 5 days each week throughout the entire 10 weeks of the experiment. The RBBO feeding dose was selected from the effective dose presented in our previous report [26]. Groups 3 and 4 were injected with 100 mg/kg body weight of DEN and 40 mg/kg body weight of DMH on the date stated in Figure 1 to initiate liver and colon carcinogenesis, respectively. Body weight, food consumption, and water intake were recorded twice weekly during the course of the experiment. At indicated times, rats were euthanized with anesthesia via the administration of isoflurane. This was performed to collect blood for the assessment of alanine aminotransferase (ALT) and aspartate aminotransferase (AST) levels in the serum using an automated analyzer provided by the Small Animal Hospital, Faculty of Veterinary Medicine, Chiang Mai University. Livers of the rats were then dissected and separated into two portions, one was flash frozen for molecular analysis and the other was fixed in 10% phosphate-buffered formalin for use in immunohistochemistry studies. The colons of half of the rats in each group (*n* = 8) were collected and placed in formalin fixative, while the colons of the remaining rats (*n* = 8) were washed with 0.9% normal saline solution and then longitudinally cut into segments that were placed flat on glass plates. Colonic mucosa cells were scraped off onto glass slides, collected in 1.5 mL tubes. They were then maintained at −80 °C. Moreover, feces samples were freshly collected from the anuses of the rats, placed in microcentrifuge tubes, and immediately kept at −80 °C.

### 2.4. Determination of Preneoplastic Lesions in Colon and Liver Tissues

Methylene blue staining was used to evaluate the colonic aberrant crypt foci (ACF). By filling the colon with 10% formaldehyde in phosphate-buffered saline (pH 7.4), it was enlarged and fixed. The colon was then sliced longitudinally and divided into three segments: rectum, proximal, and distal. The flattened colon was stained with 2% methylene blue for 1 min before being scored for ACF size and then examined under a light microscope at 40× magnification using Bird’s criteria [27].

Liver sections of 4 µm in thickness were immunohistochemically determined for GST-P positive foci by employing the avidin–biotin complex method described by Thumvijit et al. [28]. The number and area of GST-P positive foci that were greater than 0.20 mm^2^ were measured under a light microscope using the LAS Interactive Measurement program (Leica Microsystems (SEA) Pte Ltd. All Microscopy Teban Gardens Crescent Singapore, Singapore).

### 2.5. Immunohistochemistry of Proliferation Cell Nuclear Antigen (PCNA)

Cell proliferation biomarker in liver and colon tissue samples was examined using immunohistochemistry. The double-staining procedure for the liver tissue samples was performed using the EnVision Doublestain system. Liver slices were stained immunohistochemically with anti-PCNA antibody (Biolegend, San Diego, CA, USA) and anti-rat GST-P antibody, according to the manufacturer’s instructions. Under a light microscope, the number of PCNA positive hepatocytes labeled in GST-P positive foci and its surrounding region was determined to be at least 1000 hepatocytes each.

For the colon tissue samples, sections were incubated overnight with monoclonal mouse anti-rat PCNA antibody. The steps that were then taken were similar to those presented in the manufacturer’s instructions according to the liver tissue method. The number of brown-staining PCNA positive cells was determined under a light microscope and reported as the relative percentage of PCNA positive cells per total cells.

### 2.6. Terminal Deoxynucleotidyltransferase (TdT)–dUTP Nick End Labeling (TUNEL) Assay

TUNEL assay is a method used for the investigation of cell apoptosis by detecting 3′-OH ends from DNA fragmentations. Apoptotic cells in liver tissue samples were detected by employing the TUNEL and GST-P double-staining method using the ApopTag Peroxidase in situ kit and the EnVision Doublestain system as described by Thumvijit et al. [28]. The number of positive cells was counted both inside and throughout the surrounding area of the GST-P positive foci. Moreover, a cross-section of the colon was examined to detect cell apoptosis by TUNEL using the ApopTag Peroxidase in situ kit, according to the manufacturer’s recommendations. The number of brown-staining apoptotic cells was determined under a light microscopic and reported as the relative percentage of TUNEL positive cells per total cells.

### 2.7. Determination of Pro-Inflammatory Cytokine Gene Expression by Quantitative Reverse Transcription Polymerase Chain Reaction (qRT-PCR)

The mRNA was extracted from defrosted liver and colonic epithelium using Purezol reagent according to the instructions presented in the user manual. Accordingly, mRNA was then synthesized to cDNA using a high-capacity cDNA reverse transcription kit, according to the manufacturer’s instructions. The qPCR amplification was carried out in the QuantStudioTM 6 Flex System (Thermo Fisher Scientific, Waltham, MA, USA) using a SensiFAST SYBR Lo-ROX Kit at 95 °C for 2 min, followed by 40 cycles at 95 °C for 5 s, 60 °C for 10 s, and 72 °C for 20 s. Gene expression was standardized to β-actin levels and measured using the 2^−∆∆ct^ technique [29]. Table 1 presents the primer lists [29].

### 2.8. Measurement of SCFA in Rat Feces

Fecal volatile acids metabolized by gut microbiota were measured using the gas chromatography (GC) technique. SCFA were extracted from the feces by employing the modified method of Calik et al. [30]. Frozen feces specimens (100 mg) were thawed and diluted 4-fold with sterile water in sterile tubes. Feces specimens were then homogenized and centrifuged for 15 min at 4 °C, 4000× *g*. The supernatant was transferred to a new tube and mixed with 200 µL ice-cold 25% metaphosphoric acid, which was then kept on ice for 30 min. The samples were centrifuged for 10 min at 4 °C at 11,000× *g* and then filtered through a 0.45-micrometer nylon filter. Samples were analyzed using a flame ionization detector and a SCION 436-GC instrument (BRUKER, Billerica, MA, USA), coupled with RestekTM RTx-1 F and Capillary columns that were 15 m in length; 0.53 mm ID, 5 µm df (Agilent Technologies, Santa Clara, CA, USA). The injector port was fixed at 250 °C. After injection, the temperature was initiated at 80 °C and increased by 15 °C/min to 200 °C where it was held for 2 min. A combination of nitrogen and helium was employed as the carrier gas. The injection volume was set at 1 μL with a total run time of 10 min and analyzed in duplicate. The amounts of acetate, propionate, butyrate, isobutyrate, valerate, and isovalerate were computed from a mixed standard curve and expressed as µ mole/g feces.

### 2.9. Analysis of Composition of Fecal Intestinal Microbiota in Rat

Bacterial profiles in the gut were analyzed using a next-generation sequencer. Firstly, frozen feces were thawed and bacterial DNA was extracted with the use of a QIAamp^®^ DNA Stool Mini Kit (QIAGEN Inc, Germantown, MD, USA) by following the manufacturer’s suggested protocol. Bacterial DNA was amplified by 16S ribosomal RNA gene (rDNA) amplicon PCR analysis, according to Klindworth et al. [31]. The reaction was carried out in 25-microliter volumes containing 5 ng/µL of DNA samples, 12.5 µL of KAPA HiFi HotStart ReadyMix containing 2.5 mM Mg^2+^ (Kapa Biosystems, Boston, MA, USA), and 1 µM of the forward and reverse primers. Primer pairs comprised forward 5′-TCGTCGGCAGCGTCAGATGTGTATAAGAGACAGCCTACGGGNGGCWGCAG-3′ and reverse 5′-GTCTCGTGGGCTCGGAGATGTGTATAAGAGACAGGACTACHVGGGTATCTAATCC-3′. The following PCR steps were performed: denaturation at 95 °C for 3 min, 25 cycles of denaturation at 95 °C for 30 s, annealing at 55 °C for 30 s, and elongation at 72 °C for 30 s, with a final extension step at 72 °C for 5 min. A GeneJET PCR Purification Kit (Thermo Scientific, Waltham, MA, USA) was used to purify the PCR products. The extracted DNA was then measured and quantified using a nanodrop 800 spectrophotometer (Thermo Scientific, Waltham, MA, USA) and by administering agarose gel electrophoresis. The PCR products were stored at −20 °C for the purposes of sequencing. A minimum amount of PCR amplicon at 400 ng of each sample was then used to establish a sequencing library. The relevant PCR amplicon products were selected for next-generation sequencing using Miseq system (Illumina, San Diego, CA, USA) by Omics Sciences and Bioinformatics Center, Chulalongkorn University. Raw data were then stored in a fastq.gz file. The sequences were processed using CLC genomic workbench software version 20.0.3 to taxonomically classify the operational taxonomic unit (OTU) that was representative of sequences with a 97% similarity cutoff value in the following database: SILVA release 132 (https://www.arb-silva.de/documentation/release-132/, accessed on 1 August 2021).

### 2.10. Statistical Analysis

All data are presented as mean ± SEM values. The Statistical Package for the Social Sciences (SPSS) version 17.0 software was used to conduct the statistical analysis (SPSS Inc., Chicago, IL, USA). One-way analysis of variance (ANOVA) was used to determine the significant differences between groups in each experiment, followed by the least significant difference (LSD) tests. Statistical significance was defined as a value of *p* < 0.05. In terms of microbial composition, the Kruskal–Wallis *H* test was established as a non-parametric statistic in order to quantify any similarities between samples.

## 3. Results

### 3.1. Effect of RBBO on Preneoplastic Lesions of Liver and Colorectal Carcinogenesis in Rats

**Figure 2:**
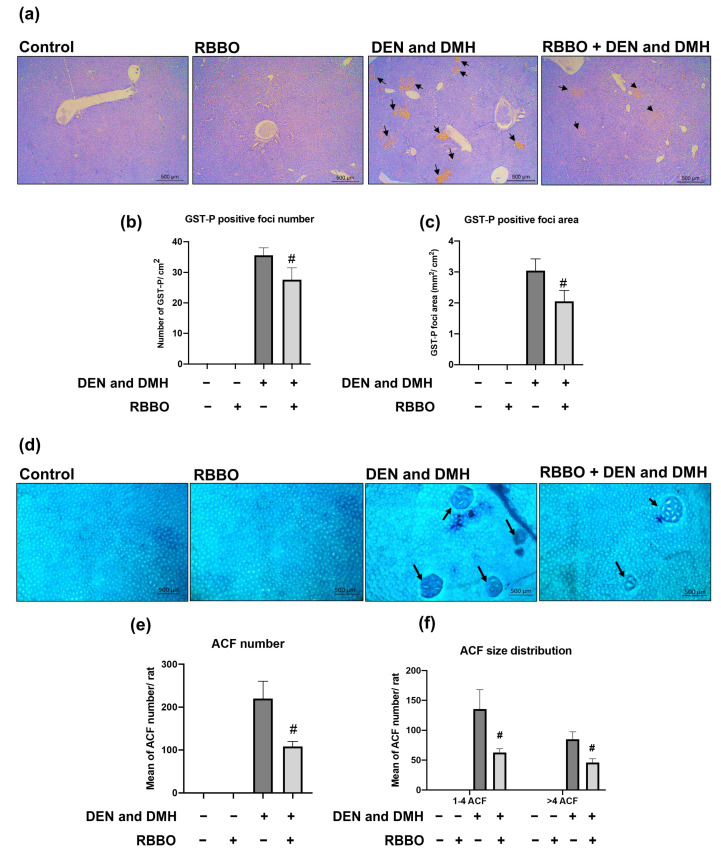
Effect of RBBO administration on hepatic GST-P positive foci and colonic ACF in DEN- and DMH-initiated rats. (**a**) Hepatic GST-P positive foci (100×), (**b**) number and (**c**) size of GST-P positive foci in liver tissues, (**d**) colonic ACF (100×), and (**e**) number and (**f**) size distribution of ACF in colon tissues. Arrows indicate GST-P positive foci and ACF. Results are expressed as mean ± SEM values (*n* = 8). # *p* < 0.05 significantly different from the DEN- and DMH-induced group. GST-P, glutathione S-transferase placental; ACF, aberrant crypt foci; RBBO, Riceberry bran oil; DEN, diethylnitrosamine; DMH, dimethylhydrazine.

The inhibitory effects of RBBO on relevant biomarkers, including GST-P and the ACF of DEN- and DMH-initiated liver and colon carcinogenicity, were examined, respectively. The administration of DEN and DMH induced toxicity in rats detected by reducing body weight and increasing serum AST and ALT levels (Table 2). They did not affect the quantities of food and water ingested by the rats, as well as the relative liver, spleen, and kidney weights of those rats (data not shown). It was indicated that these carcinogens caused liver injury in rats. Feeding of RBBO did not change body and vital organ weights, and liver function enzyme levels when compared to a negative control group, suggesting non-toxicity of RBBO to the rats. However, RBBO administration could not modulate AST and ALT levels in DEN- and DMH-induced rats. Neither nodules nor tumors were observed by H&E staining in the liver and colon tissues of DEN- and DMH-induced rats in this 10-week protocol (data not shown). Furthermore, a combined DEN and DMH injection significantly induced the development of hepatic GST-P positive foci (Figure 2a) and colonic ACF (Figure 2b). The administration of 100 mg equivalent to γ-oryzanol/kg body weight of RBBO suppressed both the number and size of hepatic GST-P positive foci in DEN- and DMH-initiated rats (Figure 2c,d). In addition, RBBO administration in carcinogen-initiated rats significantly decreased the number and size of both small foci containing 1–4 crypts per focus and large foci containing more than 4 crypts per focus, as well as those of colonic ACF when compared to the carcinogen-treated alone group (Figure 2e,f). These results indicate the potential of RBBO in the inhibition of colon- and hepatocarcinogenesis. RBBO-treated alone rats did not indicate the presence of GST-P positive foci in their liver and ACF in their colons, suggesting the non-carcinogenicity of RBBO.

### 3.2. Inhibitory Mechanism of RBBO Involved in Cell Proliferation and Apoptosis in Liver and Colon Tissues of DEN- and DMH-Initiated Rats

**Figure 3:**
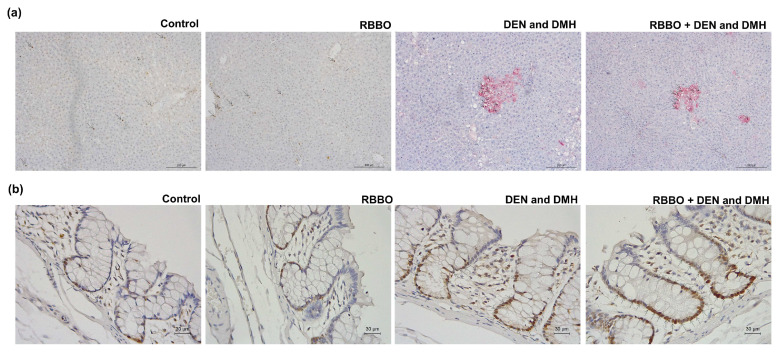
Effect of RBBO on cell apoptosis in liver and colon tissues of DEN- and DMH-induced rats. (**a**) Double-staining immunohistochemistry of apoptotic cells in hepatocytes (200×). Red areas demonstrate GST-P positive foci and arrows indicate TUNEL positive cells in both GST-P positive foci and normal areas. (**b**) Immunohistochemistry of apoptotic cells in colonocytes (400×). Arrows indicate TUNEL positive cells. RBBO, Riceberry bran oil; DEN, diethylnitrosamine; DMH, dimethylhydrazine.

To investigate whether the molecular mechanism by which RBBO inhibited preneoplastic lesions of liver and colon carcinogenicity, the biomarkers of cell proliferation and apoptosis were examined in the liver and colon tissues of DEN- and DMH-injected rats. PCNA protein representing cell proliferation was evidently verified in the liver and colon tissue samples by immunohistochemistry (Figure S1). The treatment of RBBO alone did not affect the cell proliferation of normal rat livers and colons. The number of PCNA positive cells in hepatic GST-P positive foci, those in the surrounding area, and also those in colon epithelial cells increased in the carcinogen-treated groups when compared with the negative control group. Unexpectedly, RBBO administration in carcinogen-treated rats did not alter the number of PCNA positive cells in both the liver and colon tissues when compared with those of the positive control group (Table 3). DNA fragmentation generated during apoptosis was labeled in the rat liver and colon tissues by TUNEL assay (Figure 3a,b). Rats treated with DEN and DMH revealed statistically inclined numbers of TUNEL positive hepatocytes in GST-P positive foci and in the surrounding areas, as well as in colonocytes when compared with the vehicle-injected group. Furthermore, the treatment of RBBO significantly increased the number of TUNEL positive cells in both the livers and colons of carcinogen-induced rats (Table 4). These findings indicate that RBBO could inhibit preneoplastic lesions of rat colons and hepatocarcinogenesis by the induction of cell apoptosis.

### 3.3. Effect of RBBO on the Expression of Pro-Inflammatory Genes in the Livers and Colons of Rats

**Figure 4:**
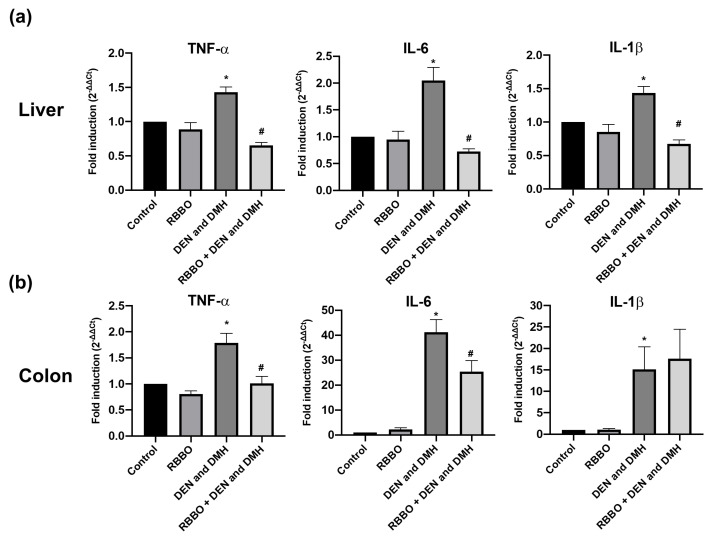
Effect of RBBO administration on mRNA levels of transcription factors and inflammatory response genes in liver (**a**) and colon (**b**) tissues of DEN- and DMH-induced rats measured by real-time PCR. Results are expressed as mean ± SEM values (*n* = 8). * *p* < 0.05 significantly different from the vehicle control. # *p* < 0.05 significantly different from the DEN- and DMH-induced alone group. TNF-α, tumor necrosis factor-alpha; IL-6, interleukin-6; IL-1β, interleukin-1beta; RBBO, Riceberry bran oil; DEN, diethylnitrosamine; DMH, 1,2-dimethylhydrazine.

An injection of DEN and DMH induced the gene expression of TNF-α, IL-6, and Il-1β in hepatocytes, and also increased all genes in the colonocytes. RBBO only did not affect the inflammatory response with regard to the unchanged mRNA levels in the colons and livers of rats. Notably, the rats administrated with RBBO significantly suppressed the expression of these induced genes including the TNF-α, IL-6, and IL-1β levels in the hepatocytes of carcinogen-treated rats (Figure 4a). Furthermore, RBBO administration significantly inhibited TNF-α and IL-6 expression in the colonocytes of carcinogen-induced rats (Figure 4b). However, RBBO treatment did not alter the gene expression of NF-κB and iNOS in DEN- and DMH-induced rats (data not shown). These findings indicate that RBBO inhibited DEN- and DMH-induced liver and colorectal carcinogenesis via the inhibition of an inflammatory response through the regulation of the expression of pro-inflammatory cytokines.

### 3.4. Effect of RBBO on Fecal SCFAs Production in Rats

The SCFA produced by gut microbiota that is associated with cancer development was determined by GC-FID. The amounts of SCFA, including acetic acid, propionic acid, butyric acid, isobutyric acid, valeric acid, and isovaleric acid, are presented in Table 5. Injections of DEN and DMH significantly decreased levels of SCFA, including butyric acid, isobutyric acid, valeric acid, and isovaleric acid, in rat feces. On the other hand, RBBO treatment significantly increased the levels of butyric acid but decreased the levels of acetate in rats administrated with or without carcinogens. Furthermore, RBBO treatment statistically restored valeric acid content in carcinogen-treated rats. These findings suggest that RBBO plays a key role in the production of bacterial metabolites of gut microbiota.

### 3.5. Effect of RBBO on Bacterial Profile in Rats

**Figure 5:**
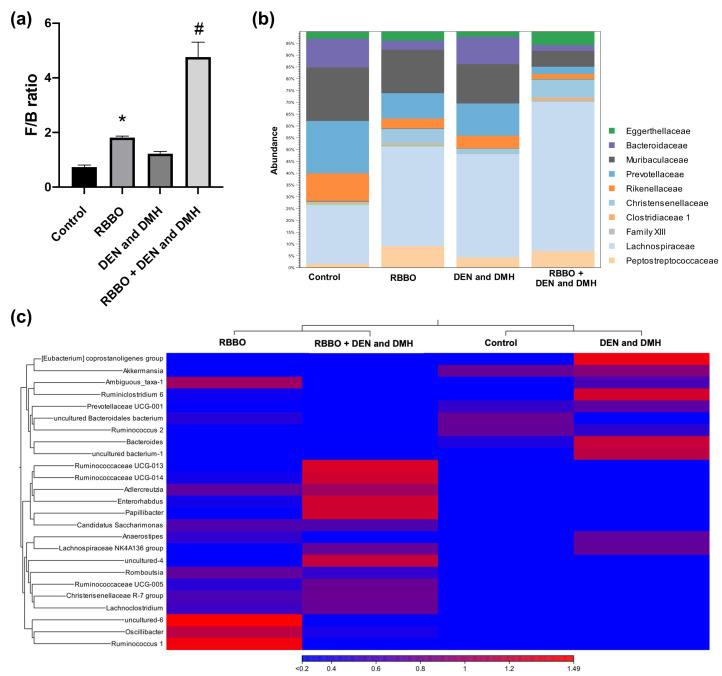
Effect of RBBO administration on the alteration of intestinal bacterial profiles. (**a**) *Firmicutes*/*Bacteroidetes* (F/B) ratio. (**b**) Relative abundance of the dominant family of each group. (**c**) Heatmap analyses of the fecal microbiota in each group. Blue-to-red shading indicates relatively lower-to-higher number of counts. Significant difference analyses were calculated using CLC Genomic workbench. * *p* < 0.05 significantly different from the vehicle control. # *p* < 0.05 significantly different from the DEN- and DMH-induced alone group. RBBO, Riceberry bran oil; DEN, diethylnitrosamine; DMH, 1,2-dimethylhydrazine.

The inhibition of the early stages of colon and liver carcinogenesis by RBBO was found to be involved with the bacterial metabolites that are present as a result of SCFA levels. Accordingly, the composition of fecal intestinal microbiota of the rats in each treatment was determined. Figure 5a depicts the ratio of dominant fecal microbiota between *Firmicutes* and *Bacteroidetes* phyla. The vehicle- and carcinogen-treated alone groups indicated an indifferent ratio for these two phyla suggesting that they were unaffected by carcinogen injections, as indicated by their bacterial profiles. In contrast, the administration of RBBO in both the vehicle- and carcinogen-treated rats resulted in an increase in the *Firmicutes*/*Bacteroidetes* (F/B) ratio by inhibiting some *Bacteroidetes* and increasing the relative abundance of *Firmicutes* when compared to the control group. This would indicate the important influence of RBBO on gut microbiota composition. The relative abundance of microbiota at the family level is demonstrated in Figure 5b. Accordingly, *Lachnospiraceae* was the predominant family of the *Firmicutes* phyla, while *Rikenellaceae*, *Prevotellaceae*, *Muribaculaceae*, and *Bacteroidaceae* represent the main families of the *Bacteroidetes* phyla in the control group, which made up 90% of the gut microbiota in normal abundance. Similar to the results of the increased *Firmicutes*/*Bacteroidetes* ratio, the RBBO treatment alone and the RBBO treatment in carcinogen-induced rats resulted in an increase in the *Lachnospiraceae* family as the outstanding gut microbiota in both of these groups, while also slightly reducing the abundance of *Bacteroidaceae* when compared to the negative and positive control groups. In order to demonstrate the detail in the modulation of gut microbiota by RBBO, we next compared the bacterial composition at the genus level using heatmap analysis as shown in Figure 5c. Heatmap visualization at the genus level demonstrated different bacteria levels in each group. The control group was associated with a slightly increased abundance of *Akkermansia*, *Bacteroidales bacterium*, and *Ruminococcus 2*, along with a lower abundance of various genera. The induction of rats by DEN and DMH was associated with an increased abundance of *Eubacterium coprostanoligenes*, *Ruminiclostridium 6*, and *Bacteroides* when compared with the control. The treatment of RBBO in carcinogen-induced rats was found to be related to an increase in genera in the *Ruminococcaceae UCG-013*, *Ruminococcaceae UCG-014*, *Adlercreutzia*, *Enterorhabdus*, *Papillibacter*, and *Lachnospiraceae NK4A136* groups, along with a decrease in the abundance of *Eubacterium coprostanoligenes*, *Ruminoclostridium 6*, and *Bacteroides* when compared to the carcinogen-induced group. The treatment of RBBO alone revealed an abundance of *Oscillibacter* and *Ruminococcus 1* as the main genera. Therefore, RBBO may alter the profile of gut microbiota that affect the bacterial metabolism resulting in changes in SCFA levels.

## 4. Discussion

Cancer development has mainly been associated with environmental chemical carcinogens. The exposure to certain food contaminants may directly contribute to liver and colon cancers. Chemoprevention using natural or synthetic agents is an alternative way for cancer therapy to inhibit, delay, or reverse each stage of carcinogenesis [32]. Several studies have reported that a diet of vegetables, fruits, whole grains, dietary fibers, micronutrients, some fatty acids, and exercise could help to protect against various types of cancers [33]. Pigmented rice possesses a higher potency in terms of anti-oxidative activities and tumor suppression than colorless rice [34,35]. This research has determined that Riceberry bran oil (RBBO), a Thai black rice cultivar, is a rich source of oleic acid, linoleic acid, γ-oryzanol, total vitamin E, and phytosterols [26], all of which have demonstrated beneficial cancer chemopreventive properties against DEN- and DMH-induced liver and colorectal carcinogenesis in rats. However, RBBO did not injure the liver as indicated by unchanged AST and ALT levels. In a previous study, neither significant effects on mortality nor pathological abnormalities were reported with the oral administration of γ-oryzanol obtained from rice bran extract at doses of 1000 and 2000 mg/kg body weight/day in Sprague–Dawley rats in a repeated-dose 90-day oral toxicity test that followed the Organization for Economic Co-operation and Development guidelines [36]. Consequently, RBBO was considered safe for consumption in rats. A study on the bioavailability of rice bran oil in rats by Fujiwara and his group demonstrated that γ-oryzanol is readily absorbed into the blood via the portal vein system and was subsequently raised to the highest concentration in the plasma after four hours of oral administration. Accordingly, it was then distributed to each organ in its original form. Ultimately, it was rapidly metabolized in the body as ferulic acid, triterpene alcohols, and phytosterols, but its intact form could still facilitate a range of physiological functions [37].

Gastrointestinal and hepatobiliary tracts that are routinely exposed to a variety of carcinogens via dietary contaminants lead to the most found cancer formation. The established model using DEN and DMH as the initiators to develop the early stages of liver and colon carcinogenesis was performed as a tool for exploring cancer chemopreventive agents [38]. DEN is a standard carcinogen that is routinely employed in rodent models in the study of hepatocarcinogenesis in order to induce preneoplastic lesions or liver tumors that have been implicated in incidences of liver cancer in humans [39]. DMH is a colon carcinogen that has been commonly used to study chemically induced colorectal carcinogenesis in rodent models caused by DNA methylation of colonic epithelial cells in the proliferative compartment of crypts, which can then lead to hyperproliferation and apoptosis resistance [40]. The exposure of these carcinogens caused the changes in xenobiotic-metabolizing enzymes, hepatic DNA adducts, and liver damage [38]. GST-P positive foci shaped in rat livers are recognized be preneoplastic lesions of liver cancer [41], while ACF are representative of a group of abnormal tube-like glands in the linings of the colon and rectum and can be used as a biomarker for colon carcinogenesis. These lesions are the earliest indicators of change in the development of colon cancer and have been detected in both rodents and humans [27]. However, DMH synergistically augmented DEN-induced preneoplastic lesions through the activation of xenobiotic-metabolizing enzymes in the livers of rats [25]. Therefore, the hepatocarcinogenicity of the combined administration of DEN and DMH in this study might be stronger than the single carcinogen-treated models. Our results have determined that RBBO displayed chemopreventive properties in DEN- and DMH-initiated rats indicated by the suppression of both hepatic GST-P positive foci and colonic ACF in rats. This outcome was consistent with previous studies which reported that the methanolic extract of purple rice bran containing high γ-tocotrienol could inhibit GST-P positive foci formation in the livers of DEN-induced rats [29]. Furthermore, the oral administration of δ-tocotrienol also suppressed ACF, polyps, and colon cancer in azoxymethane-induced colorectal carcinogenesis in rats [42]. Iqbal, J. and his group also suggested that the tocotrienol-rich fraction from rice bran oil could suppress hepatocarcinogenicity of DEN and 2-acetylaminofluorene in rats by modulation of hepatic GST activity [43], suggesting that the chemopreventive effect of RBBO might be mediated, at least in part, through the alteration of the carcinogen metabolism. In addition, tumor mass was decreased in transplanted BALB/c mice that had been fed a standard diet supplemented with 0.2% of γ-oryzanol [44]. However, the consumption of a diet supplemented with 0.3 to 2% of phytosterol influenced the morphology of colonic epithelial cells, which are crucial preneoplastic processes involved in colon carcinogenesis and may lead to a lower risk of cancer [45]. Hence, the prominent cancer chemopreventive ingredients in RBBO would likely be γ-oryzanol, phytosterols, and tocotrienols.

Sustaining proliferative signaling, resisting cell death, and tumor-promoting inflammation are all indicated as hallmarks of cancer [45]. RBBO administration significantly increased apoptosis cells in liver and colonic tissues. Furthermore, it also significantly suppressed the gene expression of TNF-α, IL-6, and IL-1β in the hepatocytes and colonocytes of DEN- and DMH-induced rats. Inflammation is a crucial factor that has been frequently linked to the development and progression of cancer. It can occur before cellular transformation or be directly exhibited in the tumor microenvironment leading to the promotion of tumor development [46]. An overexpression of pro-inflammatory cytokines, including TNF-α, IL-6, IL-1β, iNOS, and COX-2, in the colon tissue of rats was found after DMH injections [47]. Furthermore, DEN administration also increased liver TNF-α and IL-1β expression levels in rats [48]. Similarly, a previous study has revealed that the consumption of a diet containing rice bran oil could suppress TNF-α and IL-6 secretion in isolated bone marrow-derived macrophages of mice [49]. Recent studies have demonstrated a relationship between inflammation and apoptosis that activate signaling pathways through their mutual protein molecules. Fas/FasL has a dual function depending on engagement of the death receptors with their cognate ligands. Fas/FasL is a well-known death factor that induces apoptosis in a caspase-8-dependent manner. By contrast, it can activate transcriptional processes that result in NF-κB or AP-1-dependent pro-inflammatory cytokine expression [50]. Therefore, RBBO may drive the signal transduction associated with the Fas/FasL shift to apoptosis through the inflammatory pathway as a result of increased apoptosis and decreased pro-inflammatory expression to inhibit carcinogenesis. The results of previous studies support that the treatment of γ-tocopherol resulted in apoptosis induction for human colon cancer cell lines through the activation of caspase-3, -7, and -8 [51]. Moreover, α- and γ-tocotrienol induced the apoptosis of rat hepatoma dRLh-84 cells via DNA fragmentation, as well as the activation of caspase-3 and caspase-8 [52,53]. These findings suggest that the inhibition of preneoplastic lesions in the liver and the colorectal tissues of carcinogen-initiated rats by RBBO could be caused by the modulation of cell apoptosis and other relevant anti-inflammatory properties.

Previously published data have indicated that intestinal bacteria have been implicated in cancer development. Firmicutes and Bacteroidetes are the main phyla that are highly represented in more than 90% of a thousand different bacterial species found in the intestinal tract [54]. The alteration of gut microbiota in both composition and function has been associated with several pathological conditions such as inflammatory bowel disease, obesity, and the onset of colorectal cancer [55]. The modulation of gut microbiota may be an alternative approach for cancer prevention. Unexpectedly, the results of our experiments indicate insignificant differences in F/B ratios in comparisons between the control group and the carcinogen-induced alone group with regard to the duration of carcinogen injections and the stage of carcinogenesis. Sun and colleagues reported a decreased abundance of Firmicutes and a significant abundance of Bacteroidetes after subcutaneous injections of DMH at a dose of 20 mg/ kg body weight once a week for six consecutive weeks to establish an ‘adenoma–carcinoma sequence’ in mice after 26 weeks of the experiment [56]. Consequently, the frequency and number of carcinogen injections over the 10 weeks of our experiment did not result in changes in the composition of gut microbiota. Interestingly, an increase in the F/B ratio in the feces of RBBO-administrated rats was related to an increase in fecal butyrate content. Butyrate, one of the SCFA produced by the large intestinal bacteria, has been reported for its physiological function as an energy source in the growth and differentiation of human colonocytes [57]. It has also been found to be involved in numerous anti-carcinogenic actions, such as anti-inflammatory immune response, cell cycle arrest, and apoptosis induction in colon cancer, through the inhibition of histone deacetylase and by attenuating an inflammatory response in the liver [58]. The administration of RBBO in rats increased certain Firmicutes, including Lachnospiraceae, Ruminococcaceae UCG-104, and Lachnospiraceae NK4A136, which are known to be butyrate-producing bacteria [59]. These results confirm the significance of the production of butyrate by the phyla Firmicutes and their chemopreventive properties through the promotion of RBBO. Furthermore, an increase in the Eubacterium coprostanoligenes and Bacteroides families, which is evidence of colorectal cancer development [60,61], was found after DEN and DMH induction in rats. However, these levels were reduced in the RBBO-treated rats suggesting the anti-carcinogenic properties of RBBO. Moreover, conjugated linoleic acids, which were derived from the metabolism of linoleic acid in rice bran oil by probiotic bacteria, possessed anti-inflammatory and cancer preventive properties [62] while also inhibiting the growth of preneoplastic lesions of colorectal carcinogenesis in rodent models [63]. Therefore, these findings support the chemopreventive role of RBBO on the liver and in cases of colorectal carcinogenesis in rats. Because this cancer preventive activity was observed in only one dose of RBBO, the multi-dose experiment of RBBO needs to be further investigated. With regard to the advantages associated with rice bran consumption in humans, recent studies on the influence of rice bran or rice bran oil on the human gut microbiome indicate a promising potential for its application in cancer prevention. However, it is essential to highlight that increasing rice bran consumption may potentially have adverse effects. Rice bran consumption can result in a small increase in the synthesis of some bile acids, which can then result in the promotion of cancer [64]. In addition, rice bran was found to contain trace quantities of carcinogenic inorganic arsenic, which would likely be traced to the environmental contamination of the water used in the growing of rice [65]. However, the chemopreventive impact of rice bran on cancer should balance these adverse effects provided that the recommended dose of rice bran and rice bran oil is up to 30 grams per day [66].

## 5. Conclusions

**Figure 6:**
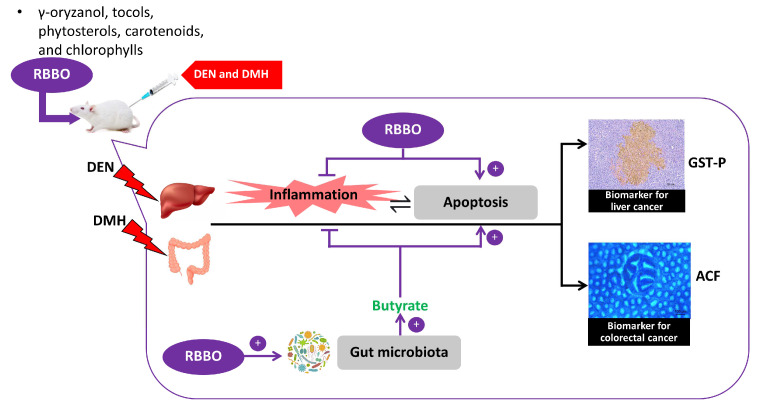
Summary of the mechanism of the action of RBBO as a chemopreventive agent on liver and colorectal carcinogenesis in rats.

These findings indicate the chemopreventive potential of RBBO on the liver and colorectal carcinogenesis induced by DEN and DMH along with involvement in the molecular inhibitory mechanism (Figure 6). The enriched bioactive compounds in RBBO, such as γ-oryzanol, phytosterols, and γ-tocotrienol, could inhibit an inflammatory response, delay cell cycle, and affect the alteration of gut microbiota, particularly in the SCFAs that are produced by bacteria. This is evidence of the regulation of cancer-related inflammation, as well as the induction of cell apoptosis, resulting in the inhibition of preneoplastic lesion formation in the livers and colons of rats. RBBO might be a promising source of valuable chemopreventive agents for either cancer prevention or treatment. The outcomes of this study point to the ever-increasing health benefits that can be attributed to RBBO in the prevention of carcinogenesis.

## Figures and Tables

**Table 1:** Primer sequences of qRT-PCR.

Genes	Forward Primer (5′-3′)	Reverse Primer (5′-3′)
tumor necrosis factor-alpha (TNF-α)	AAATGGCCCTCTCATCAGTCC	TCTGCTTGGTGGTTTGCTACGAC
Interleukin-6 (IL-6)	TGATGGATGCTTCCAAACTG	GAGCATTGGAAGTTGGGG TA
Interleukin-1 beta (IL-1β)	CACCTCTCAAGCAGAGCACAG	GGGTTCCATGGTGAAGTCAAC
inducible nitric oxide synthase (iNOS)	CAGGTGCTATTCCCAGCCCAACA	CATTCTGTGCAGTCCCAGTGAGGAA
nuclear factor kappa B (NF-κB)	GGCATGCGTTTCCGTTACAA	TGATCTTGATGGTGGGGTGC
β-actin	ACAGGATGCAGAAGGAGATTAC	AGAGTGAGGCCAGGATAGA

**Table 2:** Effect of RBBO on body weight and liver function test in rats.

Treatment	Final Body Weight (g)	Liver Function Test (Unit/L)	
		AST Activity	ALT Activity
Control	397.0 ± 6.6	60 ± 1.7	27 ± 1.4
RBBO	387.0 ± 3.7	67 ± 6.1	27 ± 3.5
DEN and DMH	369.4 ± 10.0 *	75 ± 2.8 *	40 ± 2.5 *
RBBO + DEN and DMH	352.5 ± 10.6	81 ± 4.8	49 ± 2.9

Values are represented as mean ± SEM values (*n* = 8). * Significantly different from the control group (*p* < 0.05). RBBO, Riceberry bran oil; DEN, diethylnitrosamine; DMH, dimethylhydrazine; AST, aspartate aminotransferase; ALT, alanine aminotransferase.

**Table 3:** Effect of RBBO on cell proliferation in rat liver and colon tissues.

Treatment	Number of PCNA Positive Cells in Hepatocytes		% Relative (PCNA + Cells/Total Colonocytes)
	PCNA^+^/1000GST-P^+^ Cells	PCNA^+^/1000 Surrounding Cells	
Control	ND	5.81 ± 1.39	15.31 ± 6.00
RBBO	ND	4.69 ± 2.13	15.52 ± 4.00
DEN and DMH	47.29 ± 7.98	13.58 ± 5.38 *	46.87 ± 5.38 *
RBBO + DEN and DMH	45.10 ± 8.29	12.75 ± 3.68	42.68 ± 3.68

Values are represented as mean ± SEM values (*n* = 8). * Significantly different from the control group (*p* < 0.05). ND, non-detectable; RBBO, Riceberry bran oil; DEN, diethylnitrosamine; DMH, dimethylhydrazine.

**Table 4:** Effect of RBBO on cell apoptosis in rat liver and colon tissues.

Treatment	Number of TUNEL Positive Cells in Hepatocytes		% Relative (TUNEL + Cells/Total Colonocytes)
	TUNEL^+^/1000GST-P^+^ Cells	TUNEL^+^/1000 Surrounding Cells	
Control	ND	21.47 ± 4.67	43.89 ± 8.57
RBBO	ND	26.85 ± 2.73	44.72 ± 7.48
DEN and DMH	79.86 ± 3.93	56.61 ± 5.38 *	53.92 ± 3.61 *
RBBO + DEN and DMH	91.32 ± 4.97 #	73.28 ± 6.02 #	64.84 ± 4.37 #

Values are represented as mean ± SEM values (*n* = 8). * Significantly different from the control group (*p* < 0.05). # Significantly different from the DEN and DMH group (*p* < 0.05). ND, non-detectable; RBBO, Riceberry bran oil; DEN, diethylnitrosamine; DMH, dimethylhydrazine.

**Table 5:** Effect of RBBO administration on the SCFA levels in the feces of DEN- and DMH-induced rats.

SCFA (µ mole/g Feces)	Treatment			
	Control	RBBO	DEN and DMH	RBBO + DEN and DMH
Acetate	46.34 ± 3.53	36.44 ± 6.20 *	42.19 ± 4.92	39.72 ± 2.37 ^#^
Propionate	7.41 ± 1.31	6.64 ± 3.61	7.78 ± 2.29	7.36 ± 0.72
Butyrate	1.65 ± 0.11	2.15 ± 0.76 *	1.16 ± 0.22 *	2.09 ± 0.33 ^#^
Isobutyrate	10.77 ± 2.91	6.64 ± 2.64 *	8.38 ± 1.06 *	10.63 ± 2.59
Valerate	0.84 ± 0.22	0.74 ± 0.23	0.58 ± 0.10 *	1.05 ± 0.43 ^#^
Isovalerate	2.48 ± 0.30	2.44 ± 0.43	1.64 ± 0.37 *	1.97 ± 0.66

Results are expressed as mean ± SEM (*n* = 8) values. * *p* < 0.05 significantly different from the vehicle control. # *p* < 0.05 significantly different from the DEN- and DMH-induced alone group. SCFA, short-chain fatty acids; RBBO, Riceberry bran oil; DEN, diethylnitrosamine; DMH, 1,2-dimethylhydrazine.

## Data Availability

All data generated or analyzed during this study are included in this published article.

## Supplementary Materials

The following supporting information can be downloaded at: https://www.mdpi.com/article/10.3390/cancers14184358/s1, Figure S1: Effect of RBBO on cell proliferation in liver and colon tissues of DEN- and DMH-induced rats. (a) Double-staining immunohistochemistry of PCNA positive cells in hepatocytes (200×). Arrows indicate PCNA positive cells in GST-P positive foci. (b) Immunohistochemistry of PCNA positive cells in colonocytes (40×). Arrows indicate PCNA positive cells.

## Abbreviations

ACF	aberrant crypt foci
ALT	alanine aminotransferase
AP-1	activator protein 1
AST	aspartate aminotransferase
BW	body weight
Ccnd1	cyclin D1
c-JUN	Jun proto-oncogene
COX-2	cyclooxygenase-2
cDNA	complementary deoxyribonucleic acid
DAB	3,3′-diaminobenzidine
DEN	diethylnitrosamine
DMH	1,2-dimethylhydrazine
DNA	deoxyribonucleic acid
FasL	Fas ligand
F/B	*Firmicutes*/*Bacteroidetes*
FID	flame ionization detector
GC	gas chromatography
GST-P	glutathione *S*-transferase placental form
H&E	hematoxylin and eosin
HFD	High-fat diet
IBD	inflammatory bowel disease
IL-1β	interleukin-1 beta
IL-6	interleukin-6
iNOS	inducible nitric oxide synthase
i.p.	intraperitoneal
min	minute
mRNA	messenger ribonucleic acid
MUFAs	monounsaturated fatty acids
NF-κB	nuclear factor kappa B
PCNA	proliferation cell nuclear antigen
PCR	polymerase chain reaction
PUFAs	polyunsaturated fatty acids
qRT-PCR	quantitative reverse transcription polymerase chain reaction
RBBO	Riceberry bran oil
RNA	ribonucleic acid
s.c.	subcutaneous
SCFA	short-chain fatty acids
sec	second
TNF-α	tumor necrosis factor-alpha
TUNEL	terminal deoxynucleotidyltransferase–deoxyuridine triphosphate nick end labeling
