# Supplementary figures and images for: Enriched Riceberry Bran Oil Exerts Chemopreventive Properties through Anti-Inflammation and Alteration of Gut Microbiota in Carcinogen-Induced Liver and Colon Carcinogenesis in Rats
Source: Cancers (Basel). 2022 Sep 7;14(18):4358. doi: 10.3390/cancers14184358 (PMC9496912; doi:10.3390/cancers14184358)

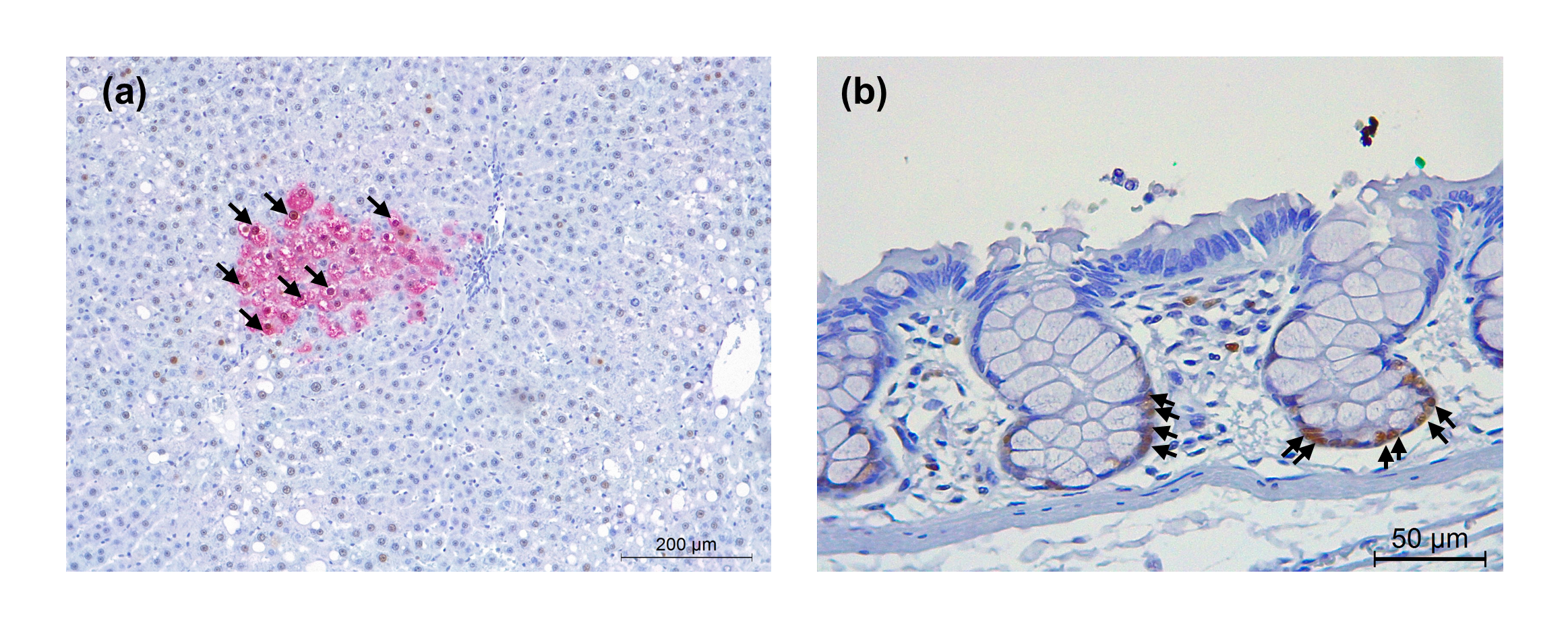

Supplement: Supplementary file 1 [file cancers-14-04358-s001.zip › cancers-1815951-supplementary Figure S1.TIF]
